# Editorial: The impact of inflammation on the oral microbiome

**DOI:** 10.3389/froh.2023.1293228

**Published:** 2023-09-22

**Authors:** Chun-Teh Lee, Loreto Abusleme, Thomas E. Van Dyke

**Affiliations:** ^1^ Department of Periodontics and Dental Hygiene, The University of Texas Health Science Center at Houston School of Dentistry, Houston, TX, United States; ^2^ Department of Pathology and Oral Medicine, Faculty of Dentistry, University of Chile, Santiago, Chile; ^3^ Laboratory of Oral Microbiology and Immunology, Faculty of Dentistry, University of Chile, Santiago, Chile; ^4^ Applied Oral Sciences, The Forsyth Institute, Cambridge, MA, United States

**Keywords:** periodontitis, microbiota, host-microbe balance/interaction, immune responce, lipid, race

**Editorial on the Research Topic** The impact of inflammation on the oral microbiome

The oral cavity is an open environment with complex microbiota eliciting sophisticated host-microbe interactions. Dysregulated host responses and microbial dysbiosis are the major etiological factors of biofilm-induced inflammatory oral diseases characterized by tissue inflammation and destruction. The relationship between shifts in the microbiome, changes in inflammatory responses, and disease progression has been investigated over decades but remains to be further clarified. This Research Topic includes five articles discussing inflammation and oral microbiome associations.

The role of lipids in periodontal inflammation and oral microbiota is relatively unexplored. In Wzatek et al. “Oral Neutrophil Free Fatty Acid Receptors Expression May Link Oral Host and Microbiome Lipid Metabolism”, authors collected oral polymorphonuclear neutrophils (oPMN) from oral saline rinse of 20 periodontally healthy individuals and measured oPMN surface expression of receptors, FFAR2 [binding bacteria-derived short chain fatty acids (SCFA)], FFAR4 [binding long chain fatty acids (LCFA), eicosapentaenoic acid (EPA), and docosahexaenoic acid (DHA)], and ERV1 [binding resolvin E1 (RvE1)] by flow cytometry. As the first-line defenders at the gingival sulcus, oPMN directly interact with biofilms. In the collected oPMN, 60% expressed ERV1 and FFAR2, and 10% expressed FFAR4, with no sex differences. Both females and males had higher expression levels of ERV1 compared to FFAR2 and FFAR4. Generally, expression levels of ERV1 and FFAR2 had a positive correlation. These findings indicated that lipids, including SCFAs, LCFAs, and RvE1, potentially can modulate periodontal inflammation by binding oPMN, and then the changes in host response can induce microbiota shifts.

Lee and Tribble, “Roles of Specialized Pro-Resolving Mediators (SPMs) and Omega-3 Polyunsaturated Fatty Acids (ω-3 PUFAs) in Periodontal Inflammation and Impact on Oral Microbiota”, reviewed the potential interactions between lipids and oral bacteria investigated from *in vitro*, preclinical and clinical studies. SPMs, a class of lipid mediators derived from ω-3 or ω-6 PUFAs, can induce the resolution of inflammation and promote local tissue homeostasis by binding receptors on immune cells. In humans, SPMs are derived from ingesting dietary ω-3 PUFA and have been identified in many specimens, including milk, serum, lymphoid tissue, saliva, gingival crevicular fluid, and gingiva. The host-modulatory effects of SPMs on periodontal tissues can drive changes in the taxonomic composition of the oral microbiota. Some *in vitro* studies demonstrated that ω-3 PUFAs have antimicrobial properties that potentially can influence the oral microbiota. Studying the relationship between host SPM production and microbiome-SPM modification has the potential to unveil new diagnostic markers of inflammation in periodontitis and discover microbial-derived bioactive therapeutics to modulate immune responses.

In Ebersole et al. “Oral Microbiome and Gingival Gene Expression of Inflammatory Biomolecules With Aging and Periodontitis”, the authors used the *Macaca mullata* (Rhesus monkey) ligature induced periodontitis model systematically characterize the relationship between host gene expression and the composition of the microbiome in young and older animals with and without disease. An important aspect of their design is the reassessment of all parameters after ligature removal during a disease resolution phase. They reported that portfolios of genes and pathways that wax and wane during the disease process are closely linked to changes in the oral bacterial complexes. These findings provide new insight into the underlying dynamics of the biological reactions in health and disease. As the authors suggest, the remaining question is the temporality of the interaction. Thus, while closely linked, the changes in host response measured by gene expression changes and accompanying microbiome changes cannot be characterized beyond their associations. In the future, temporal experiments will determine whether the changes in the microbiome induce the gene expression changes in the host or whether the host's inflammatory state drives the microbiome changes. While both likely occur, identifying specific pathways will provide new targets for personalized, rational disease therapy.

In Vaillancourt et al. “Effects of a Berry Polyphenolic Fraction of the Pathogenic Properties of *Porphyromonas gingivalis*,” the authors test the effect of a commercial berry-derived polyphenolic formulation (known as Orophenol®) on several virulence traits of the pathobiont *Porphyromonas gingivalis (Pg)*, which is strongly associated with periodontitis and several systemic conditions. This work provides relevant insights, as therapeutic strategies based on natural compounds are promising complements for managing oral diseases. First, using an *in vitro* setting, the berry polyphenolic fraction significantly reduced the growth hemolysis and inhibited the adhesion of *Pg* in a basement membrane model. Additionally, the berry polyphenolic fraction significantly decreased the activity of *Pg* proteinases and host-derived proteinases in a dose-dependent fashion. Lastly, the berry polyphenolic fraction also reduced reactive oxygen species (ROS) production in oral keratinocytes and protected these cells against the breakdown of barrier integrity. These findings lay the foundation for future studies exploring the clinical benefit of natural compounds.

In Wang et al. “Influences of Race/Ethnicity in Periodontal Treatment Response and Bacterial Distribution, A Cohort Pilot Study,” the authors investigated the response to non-surgical periodontal treatment (NSPT) of African American (AA), Caucasian (CA), and Hispanic patients (HA) and also evaluated the proportions of *Pg* and *Streptococcus cristatus* (*Sc*) in their periodontal pockets before treatment. This study provides important evidence regarding how race and ethnicity can influence the response to periodontal therapy, which has profound implications for clinical care delivery to periodontitis patients. Patients (17 AAs, 20 Cas, and 38 HAs) diagnosed with generalized periodontitis Stage II or III were examined and sampled, then received NSPT, and were clinically re-evaluated after 6 weeks. Interestingly, CA patients responded better to SRP than AAs and HAs, particularly evident on sites with the highest probing depth (>5 mm). Additionally, an increased cell count of *Pg* was observed in the HA group. However, no differences were found in the biomass of *Sc* among the three groups. These findings contribute toward a more evidence-based approach in personalizing periodontal therapy by considering race/ethnicity as an important modifier of clinical outcomes, which should be further studied in more extensive clinical studies with longer follow-ups.

In summary, this Research Topic provides an overview of the relationship between inflammation and oral microbiome and discusses the role of specific molecules, compounds, and clinical factors in host-microbe interactions. These studies' findings shed light on new research directions.

